# Environmental Predictors of Human West Nile Virus Infections, Colorado

**DOI:** 10.3201/eid1311.070506

**Published:** 2007-11

**Authors:** Jennifer L. Patnaik, Lara Juliusson, Richard L. Vogt

**Affiliations:** * Tri-County Health Department, Greenwood Village, Colorado, USA

**Keywords:** West Nile virus, epidemic, predictors, space-time, dispatch

## Abstract

To determine whether environmental surveillance of West Nile virus–positive dead birds, mosquito pools, equines, and sentinel chickens helped predict human cases in metropolitan Denver, Colorado, during 2003, we analyzed human surveillance data and environmental data. Birds successfully predicted the highest proportion of human cases, followed by mosquito pools, and equines.

The United States has experienced numerous locale-specific West Nile virus (WNV) epidemics since the infection was first identified in New York in 1999 (*1*). Since then, the Centers for Disease Control and Prevention has recommended that jurisdictions consider improving surveillance based on probability of arbovirus activity and available resources (*2*). As a result, many states have developed or enhanced environmental surveillance systems to detect WNV activity within their jurisdictions.

Tri-County Health Department (TCHD), a local public health agency in metropolitan Denver, Colorado, serves >1 million people in Adams, Arapahoe, and Douglas Counties, and spans urban and rural regions. In response to the anticipated arrival of WNV, the state of Colorado added WNV serologic testing to its existing sentinel chicken surveillance (which included serologic testing for western equine encephalitis and St Louis encephalitis), and initiated surveillance of mosquito pools, dead birds, and equines. This retrospective study aims to utilize both epidemiologic and spatial tools to determine whether the enhanced environmental surveillance system was able to predict human infections in space and time during an epidemic year of WNV activity. In addition, this study assesses whether predictability differed by urban/rural location and month of onset of human disease.

## The Study

Since 2002, healthcare providers and laboratories have been required to report patients with laboratory evidence of acute WNV infection in Colorado if testing identified WNV-specific immunoglobulin M antibodies in either cerebral spinal fluid or serum by ELISA, or blood donors with a positive nucleic acid test result. In addition to laboratory confirmation, patients had to exhibit clinical features consistent with an acute WNV infection, including encephalitis, meningitis, flaccid paralysis, or WNV fever; detailed case definitions have previously been described (*3*). Human surveillance data were downloaded from the Web-based statewide notifiable disease reporting system.

Environmental data included specimens of birds, mosquito pools, equines, and sentinel chickens that were tested for WNV. Bird oral swab specimens and mosquito pools were tested by reverse transcription–PCR or VecTest (Medical Analysis Systems, Inc., Camarillo, CA, USA) at Colorado Department of Public Health and Environment’s (CDPHE) laboratory or regional local health department laboratories. Equine specimens were tested at the Department of Agriculture and Colorado State University Veterinary Diagnostic Laboratories on a fee-for-service basis by IgM antibody capture–ELISA (MAC-ELISA) at a 1:400 dilution to eliminate false-positive results due to vaccination (*4*). Sentinel chickens were set at 3 permanent sites and tested weekly by MAC-ELISA at the Weld County Health Department laboratory. All laboratory results were reported to CDPHE and forwarded to TCHD.

A total of 408 human WNV cases were reported in Adams, Arapahoe, and Douglas Counties during 2003. TCHD’s environmental surveillance system identified 109 (50.0%) of 218 birds that tested positive for WNV, 62 (54.9%) of 113 equines that tested positive, 58 (21.7%) of 267 mosquito pools that tested positive, and 45 (12.7%) of 354 sentinel chickens that tested positive. Geographic locations were determined for all but 4 (99.0%) of the infected humans, for 96.0% of environmental species with positive results, and for 97.4% of environmental species with negative results. Human and environmental surveillance data were mapped in ArcView, version 3.3 (ESRI, Redlands, CA, USA) (Figure). The space–time relationships between human infections and environmental positive results were computed for all humans. For space, a distance threshold of 2 km was used, well within the dispersal distances identified for *Culex tarsalis* (*5*), the primary vector of WNV in Colorado. For time, the temporal threshold for environmental positive results was any time before, and up to, the time of the onset of human infection. This assumes that once an environmental positive result was documented at a particular location, WNV would remain at that location for the rest of the season. Using a script written in the Avenue programming language for ArcView 3.3, if a person was within the threshold for both space and time in relation to an environmental positive result, then we concluded that the occurrence of human infection was successfully predicted by the environmental surveillance system. Human cases that were identified as being successfully predicted and all environmental positive results are indicated in the Figure.

**Figure:**
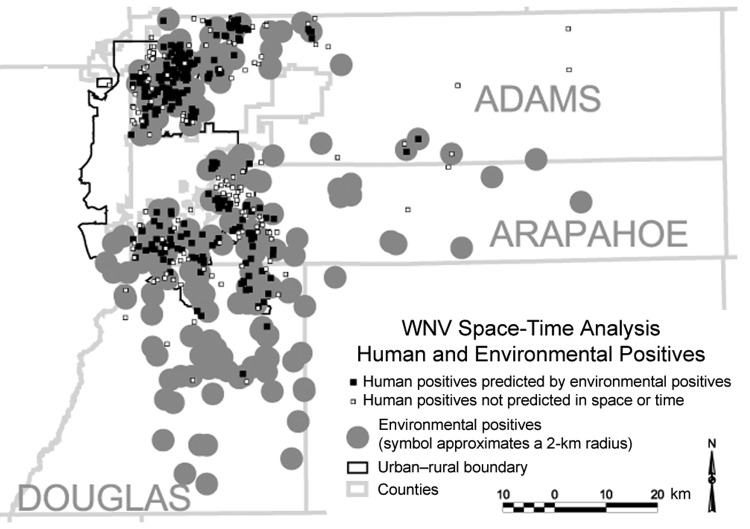
Human infections and positive environmental results, Adams, Arapahoe, and Douglas Counties, Colorado, 2003.

To determine whether environmental surveillance was more accurate at predicting human cases by urban than rural location and month of human infection onset, we calculated the proportions of human cases that were successfully predicted. Urban regions were identified as those census tracts with densities >1,600 persons per square mile (PPSM) based on Census 2000 data. In contrast to the Census Bureau’s “urban area” classification of 1,000 PPSM, this density value was derived statistically by using Colorado census tract data and did not include census blocks of low population surrounding highly built-up areas. SAS software (SAS Institute Inc., Cary, NC, USA) was used to determine measures of association by Wald χ^2^ tests with a significance level of 0.05.

Overall, the 4 types of environmental surveillance were able to predict 64.6% of all reported human cases of WNV infection (Table 1). When the 4 types of environmental specimens were analyzed separately, birds successfully predicted the highest proportion of human cases, followed by mosquito pools and equines. The accuracy rates for birds were higher in urban than in rural locations and were better in the latter half of the season. Although findings were not significant, human infections were more successfully predicted by both mosquito pools (p = 0.0874) and equines (p = 0.0782) in rural areas.

**Table 1:** Number and proportion of human infections successfully predicted by positive environmental specimens, Tri-County Health Department, Colorado, 2003

	Total no. human infections	No. (%) human Infections successfully predicted by				
		Any environmental specimen	Birds	Mosquito pools	Equines	Sentinel chickens
Region						
Urban (reference value)	292	202 (69.2)	176 (60.3)	51 (17.5)	35 (12.0)	3 (0.9)
Rural	112	59 (52.7)*	30 (26.8)*	28 (25.0)	21 (18.8)	1 (1.0)
Onset date†						
June/July	79	28 (35.4)*	17 (21.5)*	9 (11.4)*	8 (10.1)	0
August	273	196 (71.8)	157 (57.5)	54 (19.8)	41 (15.0)	4 (1.5)
September (reference value)	47	34 (72.3)	30 (63.8)	14 (29.8)	6 (12.8)	0
Total	404‡	261 (64.6)	206 (51.0)	79 (19.6)	56 (13.9)	4 (1.0)

*Significant at the p = 0.05 level. †Five human case-patients had missing onset date. ‡Four human case-patients could not be geo-located.

To validate our findings, we assessed the data in a different manner to determine how well positive environmental results predicted human infections and how well negative environmental results predicted the absence of human infections. Similar to the previous analysis, the spatial cutoff was 2 km, and the temporal threshold for environmental positive results was any time before, and up to, the time of human infection onset. Environmental negative results were expected to be followed by no human infections for at least 2 weeks. In this analysis, 50.8% of positive environmental results were followed by a human infection at some point after the environmental positive result was detected, and 86.0% of negative environmental results were followed by a lack of human cases for at least the next 2 weeks (Table 2).

**Table 2:** Proportion of human infections following positive and negative environmental specimens, Tri-County Health Department, Colorado, 2003

Specimens	No. human infections/ environmental-positive results (%)	No. human infections/ environmental-negative results (%)
All specimens	133/262 (50.8)	591/687 (86.0)
Birds	62/105 (59.0)	82/98 (83.7)
Mosquito pools	38/57 (66.7)	154/203 (75.9)
Equines	17/55 (30.9)	30/32 (93.8)
Sentinel chickens	16/45 (35.6)	325/354 (91.8)

## Conclusions

This study evaluated whether resources dedicated to environmental surveillance for the detection of WNV activity can predict human cases. More specifically, we assessed the predictability of 4 different types of environmental surveillance to identify where and when these methods were most successful in predicting human infections.

Overall, an environmental indicator preceded almost two thirds of human infections, and half of positive environmental results were followed by a human infection. Although more tests were performed for sentinel chickens and mosquito pools, birds were better predictors of human infections. Because bird surveillance depends on the public identifying and bringing in birds for testing, bird surveillance was dramatically more accurate in urban areas with high human population densities. Sentinel chicken surveillance had extremely low predictive success, which supports the subsequent decision to discontinue this surveillance method in Colorado in 2004.

This study uses a combination of epidemiologic and geographic tools to analyze WNV data spatially, temporally, and categorically. These findings would be valid when sufficient environmental samples with accurate geo-location data are submitted for testing. As with any surveillance system, environmental surveillance is likely to be more predictive during epidemic levels of virus transmission. However, the goal of state and county WNV surveillance is not to document endemic virus circulation, but rather predict increased risk for human transmission and epidemic level activity in time to initiate public notification and preventive measures.

Several other studies have illustrated the utility of dead bird testing (*6*–*8*) and dead bird clustering (*9*–*11*) in predicting human infections. Another study looked at county-level data to monitor dead birds and WNV-positive birds, as well as WNV-positive mosquito pools to predict human risk (*12*). Our study incorporates dimensions of space and time to assess the overall success of environmental surveillance at a local level. Methods used in this analysis could potentially be applied to other vectorborne diseases.
